# Spatiotemporal differentiation of *Plasmodium vivax* populations in the western Greater Mekong Subregion using a 22-SNP barcode

**DOI:** 10.1371/journal.pntd.0014472

**Published:** 2026-06-29

**Authors:** Zifang Wu, Weilin Zeng, Awtum M. Brashear, Yaming Wu, Lin Wang, Myat Thu Soe, Pyae Linn Aung, Jetsumon Sattabongkot, Myat Phone Kyaw, Zhaoqing Yang, Yaming Cao, Li Zheng, Liwang Cui, Yan Zhao

**Affiliations:** 1 Department of Immunology, College of Basic Medical Sciences, China Medical University, Shenyang, Liaoning, China; 2 Department of Pathogen Biology and Immunology, Kunming Medical University, Kunming, China; 3 Division of Infectious Disease and International Medicine, Department of Internal Medicine, Morsani College of Medicine, University of South Florida, Tampa, Florida, United States of America; 4 Myanmar Health Network Organization, Yangon, Myanmar; 5 Mahidol Vivax Research Unit, Faculty of Tropical Medicine, Mahidol University, Bangkok, Thailand; Universite de Montreal, CANADA

## Abstract

**Background:**

A high-resolution molecular tool for tracking and differentiating closely-related *Plasmodium vivax* populations is critically needed. This study aimed to develop and validate a novel single nucleotide polymorphism (SNP) barcode to monitor the progress of malaria elimination in the Great Mekong Subregion (GMS).

**Methodology/Principal findings:**

A total of 210 *P. vivax* clinical samples were collected across four time points in three international border areas: China-Myanmar border, Thailand-Myanmar border, and Bangladesh-Myanmar border. Parasites were genotyped at 36 SNPs using MassARRAY technology (Sequenom), with Sanger sequencing validation for low-efficiency loci. The complexity of infection (COI) was estimated via a maximum likelihood approach implemented in COIL, while genetic diversity metrics were computed in GenAIEx version 6.5. Population differentiation was assessed through molecular variance analysis, Mantel rank test, and pairwise *F*_ST_ estimation. Genetic structure was resolved using principal component analysis, phylogenetic analysis, and ADMIXTURE. 198 samples were successfully genotyped at 22 validated SNPs, revealing 37.9% polyclonal infections. The proportion of polyclonal infections differed significantly among the five *P. vivax* populations (*P* = 0.0001, Pearson Chi-square test, *χ*^*2*^ = 23.15), with 2020 CMB samples having the highest proportion (56.1%). The average COI was highest in BMB parasites (1.109 ± 0.007). The TMB 2018 samples exhibited the maximal nucleotide diversity (*π* = 0.342 ± 0.033) and expected heterozygosity (*H*e = 0.325 ± 0.04). The *P. vivax* populations from the western GMS showed significantly reduced genetic diversity in recent years compared to earlier timepoints (0.372 ± 0.009 vs. 0.426 ± 0.009; *P* < 0.0001, Student’s t-test). Pairwise *F*_ST_ values indicated moderate to high genetic differentiation (0.165 – 0.417) across nine population pairs, except for the temporally proximal CMB populations, which showed low differentiation. Structure analysis consistently resolved three discrete genetic clusters corresponding to CMB, TMB, and BMB parasite populations.

**Conclusions/Significance:**

This 22-SNP barcode provides a high-resolution genotyping tool capable of differentiating *P. vivax* parasite infections from the western GMS. Our data demonstrate that sustained malaria control interventions drive the fragmentation of *P. vivax* populations into genetically distinct transmission foci, creating opportunities for elimination strategies in border hotspots.

## Author summary

Despite substantial control measures, *P. vivax* has gained increasing predominance in the Greater Mekong Subregion (GMS), especially in border areas characterized by persistent transmission. Contributing factors that drive this trend include genetic diversity, human migration, and the behavior of vectors. Effectively eliminating *P. vivax* malaria in the complex border regions requires tools capable of tracking local parasite populations and transmission hotspots in real time. However, existing global genetic markers lack the resolution needed for such fine-scale surveillance. Building on prior genome-wide SNP analysis that identified high *F*_ST_ variants, we developed and validated a novel 22-SNP barcode. This tool effectively differentiates closely related parasite populations across key international border hotspots (China-Myanmar/CMB, Thailand-Myanmar/TMB, Bangladesh-Myanmar/BMB). Our study reveals significantly declining genetic diversity, particularly at the CMB, correlating with intensified control efforts. We also observed moderate-to-strong population differentiation (*F*_ST_ = 0.165–0.417), indicating fragmentation into isolated transmission foci. Critically, the detection of persistent parasite clusters underscores this barcode’s utility for identifying local clonal expansions and guiding precision interventions to eliminate residual transmission in the western GMS.

## Introduction

*Plasmodium vivax* represents the most geographically widespread human malaria parasite, with endemic transmission spanning tropical and subtropical regions of Asia, Latin America, and certain regions of Africa [1]. Despite having a lower mortality rate than *Plasmodium falciparum*, *P. vivax* poses a significant public health and economic burden due to its ability to cause relapse, subclinical infection, and early transmission to mosquitoes. These properties enable *P. vivax* to survive and transmit in more extreme climates, conferring greater resilience than *P*. *falciparum* against conventional parasite and vector control activities [2]. Moreover, *P. vivax* exhibits substantial genetic diversity and population structure, driven by factors such as host immunity, transmission intensity, and ecological adaptations [3,4]. This genetic variability affects pathogenesis, drug resistance, relapse patterns, and immunity, complicating control and elimination efforts. Therefore, understanding the genetic diversity and population structure of *P. vivax* is crucial for developing effective control strategies, including vaccines, antimalarial drugs, and surveillance systems.

In the Greater Mekong Subregion (GMS), Myanmar stands out as the most malaria-endemic country [1]. Its international borders with China, Thailand, and Bangladesh are transmission hotspots, driven by frequent cross-border population movements, limited access to healthcare services, and disparities in malaria control strategies. These conditions foster parasite persistence, facilitate genetic recombination, and promote elevated gene flow among *Plasmodium* populations, posing substantial challenges to malaria elimination across the GMS. To address these issues, advanced genetic tools are urgently needed to identify and mitigate *P. vivax* reservoirs in the region.

Various molecular tools, such as microsatellites, mitochondrial markers, short tandem repeats, and whole-genome sequencing (WGS), have been employed to characterize the genetic diversity of *P. vivax* and determine the spatiotemporal dynamics of parasite populations [5–9]. While these approaches have provided valuable insights, they commonly face limitations in terms of resolution, cost, or scalability. In recent years, several targeted genotyping approaches derived from WGS data have been developed for *P. vivax* surveillance, including amplicon sequencing (AmpSeq), molecular inversion probes (MIPs), and microhaplotypes. Amplicon‑based assays such as PvAmpliSeq and PvGAP enable high‑throughput genotyping of hundreds of markers, supporting fine‑scale population structure, recurrence classification, and tracking of drug resistance mutations [10–12]. MIPs provide scalable, cost‑effective targeted sequencing for large sample sets, with utility in defining transmission networks and copy‑number variation [13,14]. Microhaplotype panels offer enhanced resolution for estimating identity-by-descent, distinguishing closely related infections, and classifying parasite geographic clustering at subnational scales [15,16]. Collectively, these methods can achieve local‑scale geographic differentiation in some endemic regions, although their performance and resolution vary by panel design and geographic context, and most have not been rigorously validated in the GMS.

Single nucleotide polymorphisms (SNPs) have emerged as powerful molecular markers, and a small collection of SNPs (barcode) can offer high-resolution characterization of parasite populations, enabling researchers to track transmission patterns and monitor the spread of specific genotypes [17–19]. Recently, a 71-SNP barcode demonstrated strong predictive ability in identifying continent-level population clusters [20]. Although Trimarsanto et al. developed a framework identifying predictive 33-SNP, 50-SNP, and 55-SNP barcodes for determining the geographic origin of *P. vivax* infections (enabling characterization of imported cases), these barcodes remain unvalidated experimentally and are purely machine-learning based [21]. Given the substantial intercontinental differentiation of *P. vivax* populations, most barcodes developed so far can resolve the global parasite populations, but have limited power in resolving local parasite populations. For example, a 42-SNP barcode can discriminate between parasites from different continents; however, it was insufficient to differentiate parasite populations from South and Central America, different provinces of Sri Lanka, countries in Southeast Asia, and regions of the GMS [22–25].

By analyzing *P. vivax* genomic information across the GMS, we identified a panel of regionally specific SNPs, which exhibited the highest pairwise *F*_ST_ values among regional parasite populations [23]. Our study suggested that a set of 36 SNPs could effectively replicate the parasite population structure derived from genome-wide SNP analysis [23]. However, the practical utility of this *P. vivax* SNP barcode in distinguishing origins of parasite populations within the GMS requires future evaluation. In this study, we aimed to first validate this new SNP barcode as a high-resolution molecular surveillance tool for differentiating regional *P. vivax* populations using *P. vivax* clinical samples collected from Myanmar’s east and west border areas. This study aimed to further elucidate the spatiotemporal patterns of genetic diversity and population structure of *P. vivax* parasites in these border areas of the western GMS. This study not only enhanced our fundamental understanding of *P. vivax* transmission dynamics but also provided programmatically actionable data to inform and optimize evidence-based elimination strategies during the malaria elimination phase in the GMS.

## Methods

### Ethics approval

The study protocols received ethical approval from the institutional review boards of all collaborating institutions involved in the research. Specifically, approvals were granted by the ethical committees of China Medical University, China (Approval No. 2019086), the University of South Florida, USA (Approval No. Pro00036813), Mahidol University, Thailand (Approval No. TMEC11–033), and the Ministry of Health and Sports, Myanmar (Approval No. Ethics/DMR/2017/077AE/2018). Prior to participation, all individuals provided written informed consent voluntarily after receiving verbal explanations provided by trained local staff to ensure full comprehension of the study and agreement to participate. All data were de‑identified, anonymized, securely stored, and analyzed only in aggregate to protect privacy, confidentiality, and individual identities in sensitive contexts. These measures uphold rigorous ethical standards for research conducted in cross‑border and ethnically diverse settings.

### Study sites and sample collection

Clinical samples of *P. vivax* were collected from patients diagnosed with uncomplicated vivax malaria through microscopic examination at local malaria clinics or hospitals. These samples originated from three distinct border regions: 51 in 2015 and 59 in 2020 from Laiza, Kachin State, Myanmar at the China-Myanmar border (CMB) (24°45′36″ N, 97°33′48″ E); 31 in 2011 and 19 in 2018 from Mae Sod, Tak Province, Thailand at the Thailand-Myanmar border (TMB) (16°42′47″N, 98°34′29″E); and 50 in 2018 from Paletwa, Rakhine State, Myanmar at the Bangladesh-Myanmar border (BMB) (21°17′00″ N, 92°50′00″ E). Following informed consent (and assent for minors), finger-prick blood samples were spotted onto Whatman 3M filter papers, air-dried, and individually stored in plastic bags with desiccants for preservation.

### Genotyping of the 36 regionally-specific SNPs

Genomic DNA of *P. vivax* was isolated from dried blood spots on filter paper using the QIAamp DNA Mini Kit (Qiagen, Hilden, Germany). *P. vivax* parasite was verified via nested PCR amplification of the *Plasmodium* 18S rRNA gene [26], and samples with only *P. vivax* infection were used for genotyping SNPs. The 36 regionally specific SNPs identified in our earlier study were genotyped using the iPLEX SEQUENOM MassARRAY system (Sequenom, CA, USA). These SNPs are bi-allelic, non-repetitive, and functionally annotated: 29 are located in protein-coding genes, five in non-coding regions, one in a pseudogene gene, and one in an ncRNA gene [23]. Primers were designed using the MassARRAY Assay Design software (v3.1; see S1 Table), and multiplex PCR was carried out with HotStartTaq DNA Polymerase (Qiagen, CA, USA) using 12 ng of genomic DNA per reaction. Mass spectrometry data were processed using Sequenom TYPER 4.0 (Agena Bioscience, CA, USA), and genotypes were called based on spectral profiles.

To ensure assay reliability, each run incorporates two randomly chosen *P. vivax* isolates as positive controls and nuclease-free water as a negative control. Reproducibility was evaluated by: (1) repeated genotyping of control samples across multiple plates, and (2) cross-validation of multilocus samples using both MassARRAY and Taqman assays. For SNPs with suboptimal detection efficiency (chr. 1: 907060, chr. 5: 198537, chr. 9: 2162147, chr. 12: 270635), Sanger sequencing was performed on an ABI 3730XL DNA analyzer (Applied Biosystems, CA, USA). Primer sequences are listed in S2 Table.

### Data screening and quality filtration

The SNP barcode profiles exclusively comprised canonical bases (ATCG), ambiguous calls (N, indicating mixed infections), or null calls (X, representing failed or missing data). Mixed infections were inferred when chromatograms exhibited dual-peak patterns at one or more loci, matching both reference alleles. Samples were classified as negative if (1) no peaks were detectable or (2) spectral patterns deviated from control genotypes [27,28]. Population-specific minor allele frequencies (MAFs) were calculated using PLINK v1.07 [29], with regional diversity assessed through total MAFs to mitigate sampling bias. Quality filtering involves the following steps: 1) excluding samples with low detection rates (< 90%), 2) removing samples with more than 10% missing SNP calls, and 3) excluding loci with a total MAF value below 0.05 [27,28]. Additionally, we compared the clustering capabilities of two types of SNP barcodes: one consisting of SNPs with a total MAF greater than 0.05, and the other including SNPs with a total MAF less than 0.05 but having at least one population where the MAF exceeded 0.05.

### Complexity of infection (COI)

The haploid nature of blood-stage *Plasmodium* parasites means that samples exhibiting a single allele across all SNP loci are monoclonal. In each sample, the presence of at least one heterozygous site indicates that it is polyclonal. According to established criteria, infections with heterozygosity at exactly one SNP site were categorized as biclonal [30]. To ensure robustness in downstream analyses of genetic diversity and population structure, only monoclonal and biclonal infections were retained for further analysis.

The most likely number of clones per infection was estimated using genotypes at the initially targeted SNPs via a maximum likelihood approach implemented in COIL [30,31]. This method calculates the COI by evaluating the binomial probabilities of observed monomorphic versus polymorphic genotypes, while accounting for the population-level minor allele frequencies (MAFs) of the SNP panel. For enhanced accuracy, we applied the REAL McCOIL algorithm, which employs Markov Chain Monte Carlo (MCMC) simulations (10,000 iterations) to jointly estimate MAF distributions and COI [31].

### Genetic diversity

To assess the genetic diversity within populations, we employed GenAIEx version 6.5 to calculate several key metrics. Specifically, we quantified the number of haplotypes (*N*h), the number of alleles (*N*a), the number of effective alleles (*A*e), and the expected heterozygosity (*H*e) [32]. Nucleotide diversity (*π*) was estimated following the method described by Baniecki et al [19].

### Genetic differentiation analysis

*F*_ST_ quantifies the proportion of the total genetic variance attributable to differences among populations. A high *F*_ST_ value (approaching 1) indicates strong genetic differentiation between populations, suggesting restricted gene flow, genetic drift, or local adaptation. In contrast, a low *F*_ST_ value (near 0) reflects extensive gene flow and a more uniform genetic structure across populations. Using quality-controlled data, we calculated the pairwise *F*_ST_ values [33] for five populations with VCFtools [34]. To further explore the partitioning of genetic variance among the populations, an analysis of molecular variance (AMOVA) was performed using GenAlEx version 6.5. Additionally, the correlation between geographic and genetic distances was calculated using the Mantel rank test in GenAlEx version 6.5.

### Population structure analysis

Genetic relatedness patterns were examined by performing eigen decomposition of the genomic relationship matrix using PLINK 1.07 [35]. The resulting principal components were visualized in a biplot (PC1 vs PC2) generated with R ggplot2 (v3.3.0). Evolutionary relationships among *P. vivax* isolates were reconstructed through the Neighbour-Joining method based on pairwise genetic distances [36], with 1000 bootstrap replicates in MEGA7 [37]. ADMIXTURE analysis was conducted with ten independent runs for each K value (K = 1－10). Model selection was guided by minimizing cross-validation error, and the optimal K was determined by the elbow method [38].

### Statistical analyses

Statistical analyses were performed using GraphPad Prism 10.1.2. Categorical variables were analyzed using Pearson’s chi-square test or Fisher’s exact test, as appropriate. Continuous variables were compared using Student’s t-test, with data presented as mean ± SD (descriptive statistics) and mean ± SE (inferential statistics). A *P*-value < 0.05 was considered statistically significant.

## Results

### Genotyping the 36 SNP sites

A total of 210 samples were initially run on the MassARRAY platform to genotype the 36 SNP loci. To assist SNP calling and ensure accuracy, we included two positive and two negative controls. The peaks corresponding to the reference or alternative bases were consistent in the positive control samples but were either undetected or appeared as minor peaks in negative controls (S1 Fig). We reanalyzed four loci with low initial call rates by Sanger sequencing using an ABI 3730XL DNA analyzer; these loci were located at 907060 on chr. 1, 198537 on chr. 5, 2162147 on chr. 9, and 270635 on chr. 12, with initial rates of 63.8%, 46.8%, 83.0%, and 57.3%, respectively. The combined genotyping success rate (MassARRAY + Sanger) for these four loci reached 65.1%, 46.8%, 91.7%, and 63.8%, respectively. Subsequently, the loci were excluded with a combined genotyping success rate below 90%, which included 907060 on chr. 1, 198537 on chr. 5, and 270635 on chr. 12 (S1 Dataset). The overall genotyping success rate of the 33 SNPs in 210 samples was 93.7% (7085/7560 SNP calls). Meanwhile, 12 samples with more than 10% missing SNP calls were excluded from the analysis (S1 Dataset).

**Fig 1:**
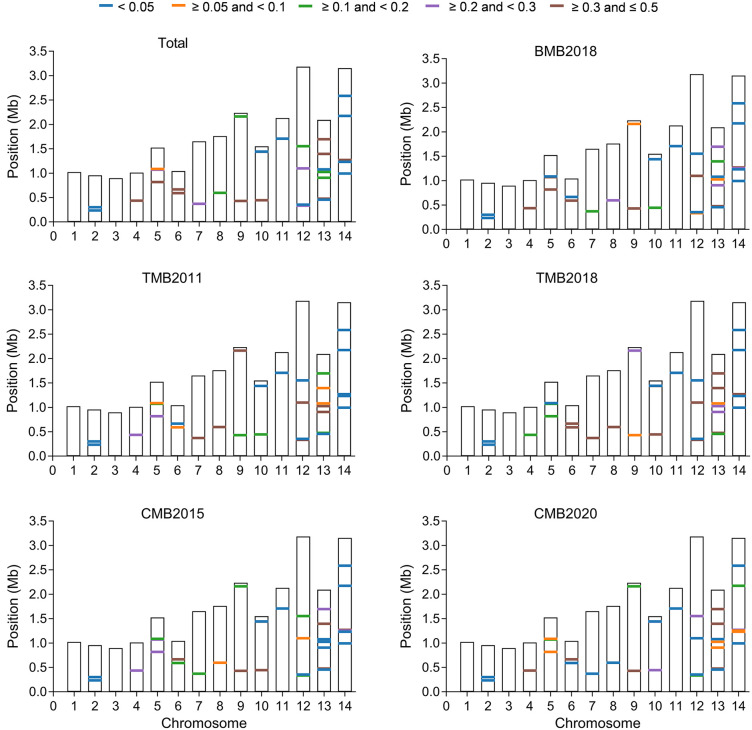
The total minor allele frequency (MAF) of 33 SNPs (Three sites with detection rates lower than 90% were excluded). The location of the 33 SNPs is illustrated on the 12 chromosomes of the *P. vivax* genome. SNPs were colored by total MAF. 2 SNPs had 0.3 ≤ MAF < 0.5, 13 SNPs had 0.2 ≤ MAF < 0.3, 5 SNPs had 0.1 ≤ MAF < 0.2, 2 SNPs had 0.05 ≤ MAF < 0.1, and 11 SNPs had MAF < 0.05.

Then, we calculated the MAF values of 33 SNPs for the remaining 198 samples. Twenty-one SNPs had an average MAF above 0.1 (Fig 1, S3 Table), while SNPs at positions 1087230 on chr. 5, 1079191 and 455190 on chr. 13, as well as 1231865 and 2173639 on chr. 14 had relatively low levels of diversity, with a total MAF of 0.071, 0.023, 0.01, 0.03, and 0.031, respectively (Fig 1, S3 Table). Based on the filtering criteria, SNPs at positions 234032, 301614 on chr. 2, 1441770 on chr. 10, 1708471 on chr. 11, 353253 on chr. 12, 1079191, 455190 on chr. 13, and 1231865, 455190, 2586551, 992879 on chr. 14 with total MAF value less than 0.05 were filtered out.

Finally, 198 samples were successfully genotyped at these 22 SNPs (Table 1). Among these, 123 infections (62.1%) exhibited monoallelicity at each nucleotide position (i.e., monoclonal), while the remaining 75 (37.9%) samples exhibited two alleles at one or more loci, indicative of polyclonal infections. Among the latter, 37 infections (18.7%) showed two alleles at a single locus (i.e., biclonal). Ultimately, 160 samples (41 from CMB2015, 44 from CMB2020, 26 from TMB2011, 12 from TMB2018, and 37 from BMB2018) with monoclonal and biclonal infections were used for population genetic analyses (Table 1).

**Table 1:** Complexity of infection in different *P. vivax* populations in the GMS.

Populations ^a^	Sequenced samples	After filtering ^b^	Polyclonal infections ^c^ (%)	Monoclonal infections ^d^ (%)	Biclonal infections ^e^ (%)	COI ± SE ^f^
**CMB2015**	51	49	9 (18.37)	40 (81.63)	1 (2.04)	1.089 **±** 0.007
**CMB2020**	59	57	32 (56.14)	25 (43.86)	19 (33.33)	1.070 ± 0.010
**TMB2011**	31	29	5 (17.24)	24 (82.76)	2 (6.90)	1.000 ± 0.000
**TMB2018**	19	14	7 (50.00)	7 (50.00)	5 (35.71)	1.000 ± 0.000
**BMB2018**	50	49	22 (44.90)	27 (55.10)	10 (20.41)	1.109 ± 0.007
**Total**	210	198	75 (37.88)	123 (62.12)	37 (18.69)	1.069 ± 0.002

a CMB, China-Myanmar border; TMB, Thailand-Myanmar border; BMB, Bangladesh-Myanmar border. ^b^ For quality filtration, SNPs with a total MAF of less than 5% and individuals with more than 10% missing SNP calls were filtered out. ^c^ Samples carried one or more polymorphic sites. ^d^ Samples carried a single allele at all positions. ^e^ Samples displayed a single polymorphic site. ^f^ COI, the most likely number of clones; SE, standard error.

### COI

The proportions of polyclonal infections differed significantly among the five *P. vivax* populations (*P* = 0.0001, Pearson Chi-square test, *χ*^*2*^ = 23.15), with the highest (56.1%) in the 2020 CMB population and lowest (17.2%) in the 2011 TMB population (Table 1). The average COI was highest in BMB (1.109 ± 0.007) and lowest in TMB (1.000 ± 0.000) (Table 1). When comparing temporal samples within a specific region, we found that the proportion in CMB was significantly higher in 2020 than in 2015 (*P* = 0.0001, Fisher’s exact test). Similarly, samples collected in TMB in 2018 had a significantly higher proportion of polyclonal infections than in 2011 (*P* = 0.0351, Fisher’s exact test). We classified the isolates into early (2011 and 2015) and recent (2018 and 2020) groups according to the major shift in malaria control strategies and the marked reduction in transmission intensity observed in the western GMS after 2015 [39–43]. This temporal stratification enabled comparisons of *P. vivax* infection complexity under contrasting intervention and transmission settings. However, no significant difference was observed among the three *P. vivax* populations sampled in the more recent years of 2018 and 2020 (*P* = 0.5125, Pearson Chi-square test, *χ*^*2*^ = 1.337). Across the GMS, both the proportion of polyclonal infections (*P* < 0.0001, Pearson Chi-square test, *χ*^*2*^ = 21.73) and the most likely number of clones (1.055 ± 0.004 vs 1.078 ± 0.005, *P* = 0.0314, Student’s t-test) were significantly higher in the recent years than in earlier years.

### Population diversity

Seventy-one unique haplotypes (44.4%) were identified among the 160 monoclonal and biclonal infections (Table 2). Parasites collected from the BMB in 2018 harbored the greatest number of haplotypes and the highest haplotype diversity (*H*d *=* 0.997). The pairwise nucleotide diversity (*π*), number of effective alleles (*A*e), and expected heterozygosity (*H*e) were highest in the vivax population from the TMB in 2018 (Table 2). At the CMB, *π* and *He* values were consistent in 2015 and 2020, with no significant differences (*P* > 0.05, Student’s t test) (Table 2). At the TMB, nucleotide diversity in parasite populations increased significantly from 0.265 in 2011 to 0.354 in 2018 (*P* < 0.0001, Student’s t test). Additionally, the values of *Ae* and *He* showed an upward trend during the same period, although no statistically significant difference was observed (Table 2). In earlier years, CMB samples had a significantly higher *π* value than the TMB samples (*P* < 0.0001, Student’s t-test), but the *H*e values were similar between the two sites (*P* = 0.5876, Student’s t test) (Table 2). Samples collected in recent years from the CMB, TMB, and BMB showed significantly different *π* values (*P* < 0.0001, one-way ANOVA), with TMB showing the highest value (0.342) and CMB the lowest (0.279) (Table 2). Collectively, the *P. vivax* populations from the western GMS exhibited substantially higher genetic diversity in earlier years than recent years (0.426 ± 0.009 vs 0.372 ± 0.009, *P* < 0.0001, Student’s t test), consistent with parasite population reduction resulting from the malaria elimination efforts rolling out in this region.

**Table 2:** Genetic diversity of *P. vivax* populations in the GMS.

Population	N	*N*h	*H*d ± SD	*π* ± SD	*N*a ± SE	*A*e ± SE	*H*e ± SE
**CMB2015**	41	21	0.912 ± 0.033	0.304 ± 0.026	1.909 ± 0.063	1.485 ± 0.064	0.297 ± 0.033
**CMB2020**	44	16	0.879 ± 0.038	0.279 ± 0.019	1.955 ± 0.045	1.399 ± 0.071	0.247 ± 0.037
**TMB2011**	26	21	0.975 ± 0.021	0.282 ± 0.019	1.955 ± 0.045	1.461 ± 0.071	0.280 ± 0.035
**TMB2018**	12	8	0.848 ± 0.104	0.342 ± 0.033	1.864 ± 0.075	1.583 ± 0.083	0.325 ± 0.040
**BMB2018**	37	35	0.997 ± 0.007	0.302 ± 0.011	1.864 ± 0.075	1.506 ± 0.080	0.293 ± 0.040
**Total**	160	71	0.973 ± 0.006	0.403 ± 0.007	2.000 ± 0.000	1.674 ± 0.062	0.383 ± 0.025

*N,* number of samples; *N*h, number of haplotypes; *H*d, haplotype diversity; *π,* pairwise nucleotide diversity; *N*a*,* number of different alleles; *A*e*,* number of effective alleles; *H*e*,* expected heterozygosity; SD*,* standard deviation; SE*,* standard error. CMB*,*China-Myanmar border; TMB, Thailand-Myanmar border; BMB, Bangladesh-Myanmar border.

### Genetic variation and differentiation

**Fig 2:**
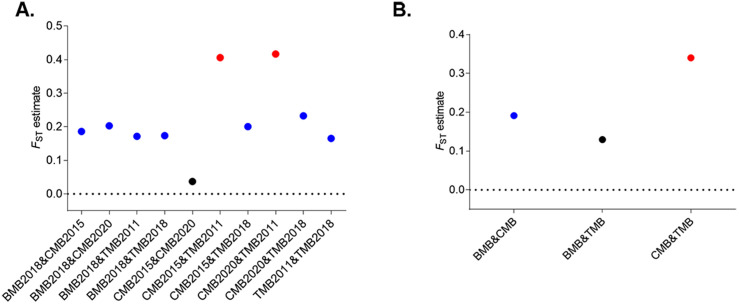
Pairwise comparison of *F*_ST_ among *P. vivax* populations in the GMS. **(A)** Pairwise comparison of *F*_ST_ among spatio-temporally specific populations. **(B)** Pairwise comparison of *F*_ST_ among regionally defined populations. Red, blue and black points represent high (*F*_ST_ ≥ 0.25), moderate (0.15 < *F*_ST_ < 0.25), and low (*F*_ST_ ≤ 0.15) levels of genetic differentiation, respectively.

AMOVA showed that the proportions of differences within and between populations were 70% and 30%, respectively (S2 Fig). Mantel tests were performed to evaluate the correlation between genetic and geographic distances. At the population level, analysis using the three sampling sites as independent units showed no significant correlation (*R²* = 0.6149, *P* = 0.410) (S3A Fig). At the individual level, a significant positive correlation was detected (*R²* = 0.2451, *P* = 0.010) (S3B Fig); however, as all isolates originated from only three fixed sites and no precise individual coordinates were available, this result should be interpreted with caution. To further explore the genetic differentiation among the *P. vivax* populations in the GMS, Wright’s fixation index *F*_ST_ was estimated for each population pair (Fig 2). Pairwise comparisons revealed the *F*_ST_ values ranging from 0.037 to 0.417 among spatio-temporally specific populations (Fig 2A). Notably, except for the two temporal CMB sample sets, which exhibited low genetic differentiation, the other nine population pairs demonstrated moderate to high genetic differentiation, with *F*_ST_ values ranging from 0.165 to 0.417 (Fig 2A). In earlier years, the populations CMB2015 and TMB2011 showed substantial genetic differentiation (*F*_ST_ = 0.406). In recent years, three pairwise comparisons — between CMB2020 and BMB2018, CMB2020 and TMB2018, as well as BMB2018 and TMB2018 — indicated moderate genetic differentiation, with *F*_ST_ values ranging from 0.174 to 0.232 (Fig 2A). When temporal heterogeneity was disregarded, the *P. vivax* populations in the CMB region had moderate to high genetic differentiation compared to those in the other two regions (*F*_ST_ = 0.191 and 0.340, respectively). In contrast, populations in the TMB and the BMB areas displayed low genetic differentiation (*F*_ST_ = 0.130) (Fig 2B).

### Population structure

**Fig 3:**
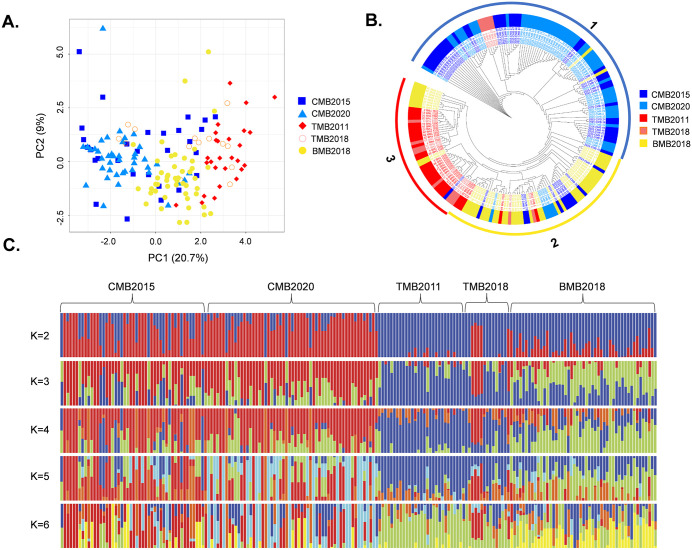
Clustering patterns of *P. vivax* isolates collected from the western GMS. **(A)** Principal component analysis. **(B)** Phylogenetic analysis of *P. vivax* isolates based on the neighbor-joining method. **(C)** ADMIXTURE analysis for K = 2－6. Vertical bars indicate individual *P. vivax* haplotypes, and colors represent individual assignments to inferred clusters. CMB, China Myanmar border; TMB, Thailand-Myanmar border; BMB, Bangladesh-Myanmar border.

To investigate the genetic structure of *P. vivax* populations from different regions within the western GMS, we applied the 22 SNPs with total MAFs above 0.05 for PCA, phylogenetic, and ADMIXTURE analyses. From the PCA, the top two principal components accounted for 20.7% (PC1) and 9% (PC2) of the total genetic variations, respectively (Fig 3A). First, parasites from the CMB, TMB, and BMB represent discernible geographic clustering with partial overlap, reflecting moderate genetic differentiation and limited residual gene flow among these cross-border regions. Despite significant declines in malaria incidence over the past decade, extensive mixing of the CMB populations collected in 2015 and 2020 was observed. Similarly, the 2011 and 2018 TMB populations also showed extensive mixing, suggesting relatively persistent parasite haplotypes in these border regions. Interestingly, the 2018 TMB *P. vivax* populations formed two separate clusters; one was mainly mixed with the earlier TMB population from 2011, while the other clustered with the CMB samples (Fig 3A). The phylogenetic tree further corroborated the findings of PCA, with the three main clusters representing parasites from the CMB, BMB, and TMB, respectively (Fig 3B).

ADMIXTURE analysis uncovered a distinct distribution pattern of *P. vivax* haplotypes, confirming genetic substructure within the western GMS parasite populations. Using the delta K method, the optimal number of sub-populations was determined to be 2 (K = 2), with the CMB populations well separated from the TMB and BMB populations (Fig 3C). The BMB population was more genetically related to the TMB populations. At K = 3 or 4, Bayesian clustering analysis further separated the parasites into three genetic groups corresponding to their three distinct geographical origins (CMB, TMB, and BMB). These results indicate that while CMB represents the most deeply divergent lineage, TMB and BMB also form geographically distinct clusters with moderate genetic differentiation (Fig 3C). Again, for the CMB and TMB populations, we did not see temporal separation of the parasites collected from the same area.

We also observed independent loci with MAF values greater than 0.05 in regional populations, including SNPs at positions 1079191 and 455190 on Chr. 13, as well as 1231865 and 2173639 on Chr. 14 (S3 Table). Given their potential in differentiating *P. vivax* populations within the GMS, we added four additional loci to our 22 SNPs to see if this would improve our ability to resolve the parasite populations from the western GMS. PCA and phylogenetic tree reconstruction with this expanded barcode revealed clustering patterns consistent with those from the original 22-SNP set (S4A and S4B Fig). However, ADMIXTURE analysis demonstrated similar resolution of parasite populations at K > 2, while it clearly separated the two temporal sample collections at the CMB (S4C Fig).

## Discussion

Genomic approaches can predict transmission intensity and may assist in tracking parasite infections and transmission networks. Given the substantial differences in selection pressures at the global level (human hosts, mosquito vectors, ecological environments, and malaria control measures), multiple genetic methods have been developed to monitor the prevalence of *Plasmodium* [19,20,44]. However, currently, there are no effective genetic tools available for differentiating *Plasmodium* populations at the local level, particularly in the GMS where vivax malaria is the predominant malaria species. This limitation hinders the monitoring of *P. vivax* transmission dynamics in this region. Previously, we utilized genome-wide SNPs to identify a set of SNPs with high *F*_ST_ values [23]. In this study, we evaluated this SNP panel for differentiating closely related *P. vivax* parasites from different regions in the western GMS, leading to the identification of a novel 22-SNP barcode with discriminatory power for spatial *P. vivax* populations in this area.

The relationship between the polyclonal infection or COI and parasite intensity is multifaceted, reflecting variations in genetic diversity and transmission dynamics [45–47]. In contrast to the high COI (88.3%) previously reported using the 42 SNPs [25], the average polyclonal proportion determined using the 22 SNPs was much lower (37.9%), but it was comparable to the results detected using the 10 microsatellite markers (30.7 – 40%) [48]. Since the samples analyzed by these two SNP combinations were all sourced from the western GMS, with some samples being partially overlapping but having non-redundant detection sites, it suggests that a higher number of SNP sites may capture greater complexity in the genetic profiles. Studies have demonstrated that the expanded distribution of SNP loci across multiple chromosomes enhances the accuracy in detecting complex *Plasmodium* infections by enabling more consistent SNP frequency calls [31,49]. Additionally, significant temporal variations were observed in both polyclonal infections and COI, with values in recent years being notably higher than those in earlier years. Our findings appear inconsistent with recent reports indicating a decline in transmission intensity, which is attributed to the rigorous implementation of malaria prevention and control strategies in the GMS [25,48]. This contradictory result may be attributed to the disproportionately high prevalence of biclonal infections, ranging from 40% to 71.1% within the polyclonal infections.

Consistent with the 42-SNP barcode, the nucleotide diversity analysis based on 22 SNPs indicated that the *P. vivax* population in the GMS exhibited moderate to high genetic diversity, with π values ranging from 0.279 to 0.342 [25]. Studies indicate that *P. vivax* populations exhibit a range of nucleotide diversity values, influenced by local transmission intensity and genetic factors. A study utilizing Illumina-based amplicon sequencing for *pvdbp*_*II*_ and *pvmsp1*_*42*_ in *P. vivax* populations across various regions of Thailand demonstrated that areas nearing pre-elimination exhibited relatively lower genetic diversity compared to highly endemic regions such as Tak Province [50]. In our study, the nucleotide diversity of *P. vivax* populations in recent years has been significantly lower than that in earlier years, which is consistent with the continuous decline in malaria cases in the GMS [51]. In addition, recent years have witnessed significant variations in nucleotide diversity across different regions, with the TMB having the highest diversity, followed by the BMB, and the CMB displaying the lowest. The low genetic diversity of *P. vivax* at the CMB highlights the effectiveness of malaria control measures implemented in recent years, particularly the effectiveness of the joint “3 + 1” malaria strategy along the CMB areas [52]. Simultaneously, it also underscores the need to reinforce malaria prevention and control measures in the border areas between Thailand and Myanmar.

While global analyses of *P. vivax* using microsatellites, antigen-encoding genes, and genome-wide SNPs have consistently revealed strong continent-scale population structures, these markers have limited power to resolve local parasite populations [53–56]. In this context, our customized 22-SNP barcode demonstrated strong discriminatory power for differentiating *P. vivax* populations across the western GMS. Although within-population genetic variation exceeded between-population (70% vs. 30%), the observed divergence among populations substantially surpassed that of other established molecular tools (e.g., 42-SNP barcode: 15%; 10-microsatellite panel: 13%) [25,48]. Concurrently, *F*_ST_ values indicated moderate to high genetic differentiation (*F*_ST_ = 0.165–0.417). Multiple molecular tools have consistently identified distinct population structures and signatures of independent evolution in *P. vivax* populations along the CMB, features that persist even when employing global genotyping panels [23,25]. Such a population divergence likely reflects intensified malaria control, creating isolated transmission hotspots that allow these parasite populations to evolve independently, as previously observed for *P. falciparum* populations in the CMB region [57]. Yet, the persistent clusters of closely related parasites in recent years have highlighted the ongoing local “clonal” transmission and gene flow within these border regions. These findings underscore the critical need for real-time surveillance to detect clonal expansion and for precision intervention strategies targeting border transmission hotspots to maintain regional *P. vivax* genotypes until elimination is achieved.

This study has several limitations that should be considered when interpreting the results. First, while the 22‑SNP barcode performs well for population‑level geographic discrimination in the western GMS, its resolution is constrained by the observed genetic variation distribution. As shown by AMOVA, approximately 70% of genetic variation occurs within populations rather than among them, limiting the precision of individual isolate assignment. In addition, the top two principal components of PCA explain less than 30% of the total variance, which may obscure subtle population substructure and lead to partial clustering overlap among groups. Second, the close genetic relationship between TMB and BMB parasites relative to CMB parasites likely reflects similarities in malaria control intensity and shared evolutionary backgrounds, rather than strict geographic proximity. This observation highlights the need to integrate intervention history and epidemiological context when interpreting population structure, rather than relying solely on genetic clustering. Third, several modern genomic approaches (including amplicon sequencing, MIPs, and microhaplotypes) have been developed for fine‑scale geographic tracking; however, their performance for discriminating *P. vivax* populations in the western GMS remains incompletely evaluated. Our panel was selected for operational simplicity, cost efficiency, and scalability in routine surveillance. Fourth, geographic distance analyses were based on sampling locations at malaria clinics, which may not precisely represent the true infection origin of individual parasites, introducing minor uncertainty into isolation‑by‑distance assessments. These limitations underscore opportunities for future improvement, including marker optimization, integration of higher‑resolution genotyping platforms, and more precise spatial sampling, to further strengthen molecular surveillance toward regional malaria elimination.

## Conclusion

The genetic diversity and distinct population structures of *P. vivax* complicate management strategies, as these factors influence transmission dynamics and the effectiveness of interventions. To assess the effectiveness of ongoing malaria control efforts at GMS regions, a genotyping tool capable of resolving parasite genetic diversity, relatedness, and transmission networks is essential. This study developed and validated a novel 22-SNP barcode for high-resolution genotyping of *P. vivax* populations in the GMS. The barcode effectively differentiates population-level genetic clusters corresponding to transnational transmission hotspots (CMB, TMB, and BMB), with declining genetic diversity and CMB-specific population fragmentation directly correlating with intensified border interventions. While this tool does not support definitive geographic prediction of random individual haplotypes, it enables robust characterization of population structure, transmission fragmentation, and local clonal expansion dynamics. These features underscore the barcode’s utility for population-based genomic surveillance and precision targeting of residual transmission foci in the western GMS.

## Supporting information

S1 Fig The mass spectrometry peaks of quality control samples were detected by using Typer 4.0 software. Each SNP locus shows one unextended primer (UEP) peak followed by two allele-specific product peaks. Dual product peaks denote a heterozygous genotype, and the three peaks for one SNP are marked with the same color. Each panel includes 20 or 16 SNPs with UEP peaks ordered by molecular weight. Peak colors are randomly assigned and not fixed across panels or reactions; color consistency between rows does not indicate identical SNPs.
(TIF)

S2 Fig Analysis of molecular variance of *P. vivax* isolates obtained from the GMS. (TIF)

S3 Fig Mantel test of *P. vivax* isolates obtained from the GMS. (A) Population-level analysis using the three sampling sites as independent analytical units. (B) Individual-level analysis based on pairwise genetic and geographic distances.
(TIF)

S4 Fig Parasite populations from the western GMS analyzed using 26 SNPs. (A) Principal coordinate analysis. The 26 SNPs showed a similar power to distinguish *P. vivax* populations as the 22-SNP barcode. (B) The phylogenetic analysis using the neighbor-joining method. (C) ADMIXTURE analysis (K = 2 – 6).
(TIF)

S1 Table 36-SNP molecular barcode and primers used in MassARRAY assay. (DOCX)

S2 Table The amplification and sequencing primer sequences used for SNP assay 1, 7, 13 and 21 on an ABI 3730XL DNA analyzer. (DOCX)

S3 Table Minor allele frequency (MAF) of *P. vivax* populations. Red font indicates that the total MAF value is less than 0.05. WC, western China; NEM, northeastern Myanmar; WM, western Myanmar; SM, southern Myanmar; WT, western Thailand.
(DOCX)

S1 Dataset Barcodes for the 210 clinical *P.* *vivax* isolates from the western GMS. The top panel shows the assays number along with its corresponding reference (REF.) or alternate allele (ALT.) and chromosome (Chr.) position. The reference allele is indicated in white, while the alternate allele is shown in golden. Polygenomic genotypes containing both alleles are labeled N and highlighted in blue, and missing SNPs are marked with X and displayed in red.
(XLSX)
