# Supplementary figures and images for: Spatiotemporal differentiation of Plasmodium vivax populations in the western Greater Mekong Subregion using a 22-SNP barcode
Source: PLoS Negl Trop Dis. 2026 Jun 29;20(6):e0014472. doi: 10.1371/journal.pntd.0014472 (PMC13340800; doi:10.1371/journal.pntd.0014472)

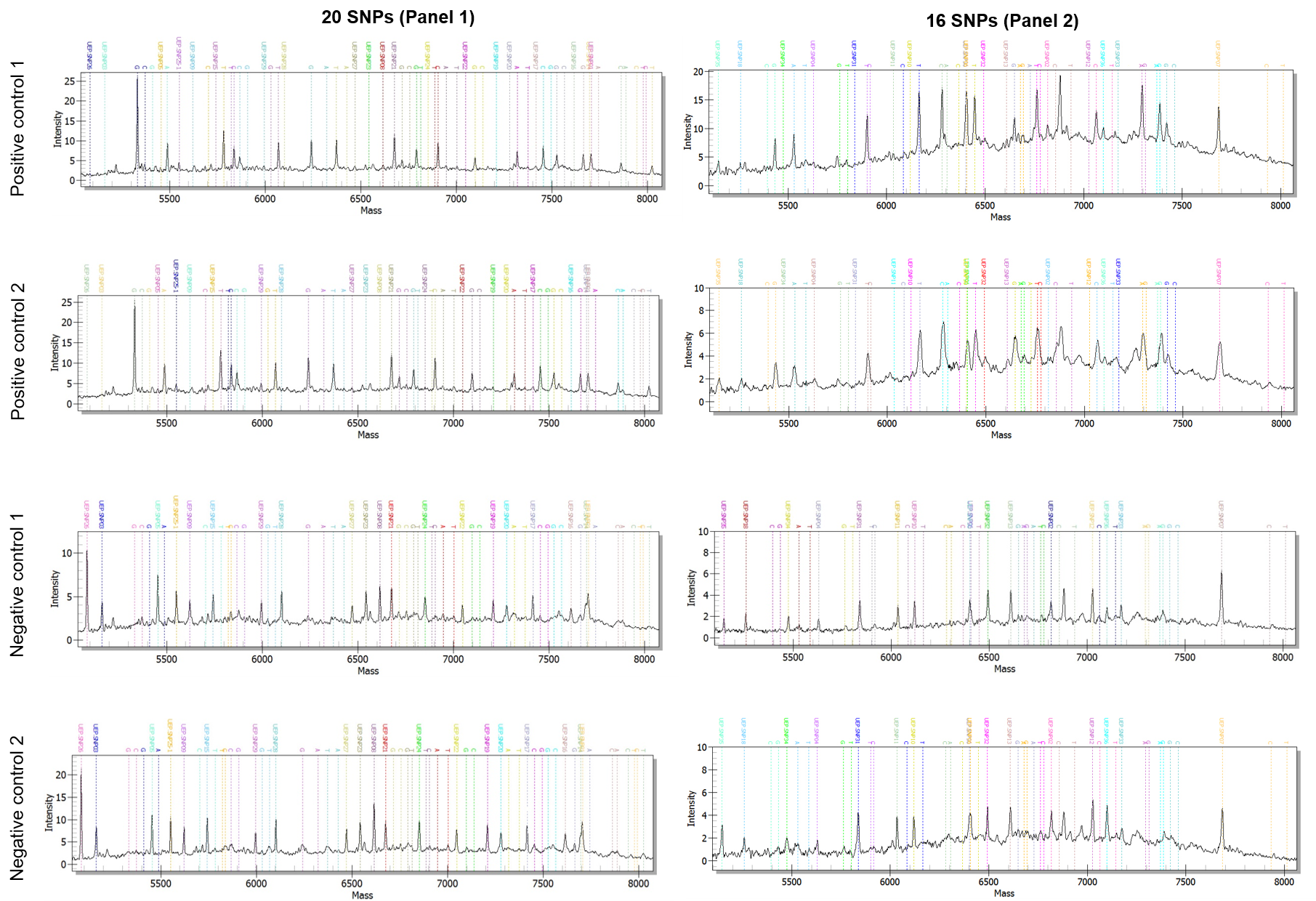

Supplement: S1 Fig — Each SNP locus shows one unextended primer (UEP) peak followed by two allele-specific product peaks. Dual product peaks denote a heterozygous genotype, and the three peaks for one SNP are marked with the same color. Each panel includes 20 or 16 SNPs with UEP peaks ordered by molecular weight. Peak colors are randomly assigned and not fixed across panels or reactions; color consistency between rows does not indicate identical SNPs. (TIF) [file pntd.0014472.s001.tif]

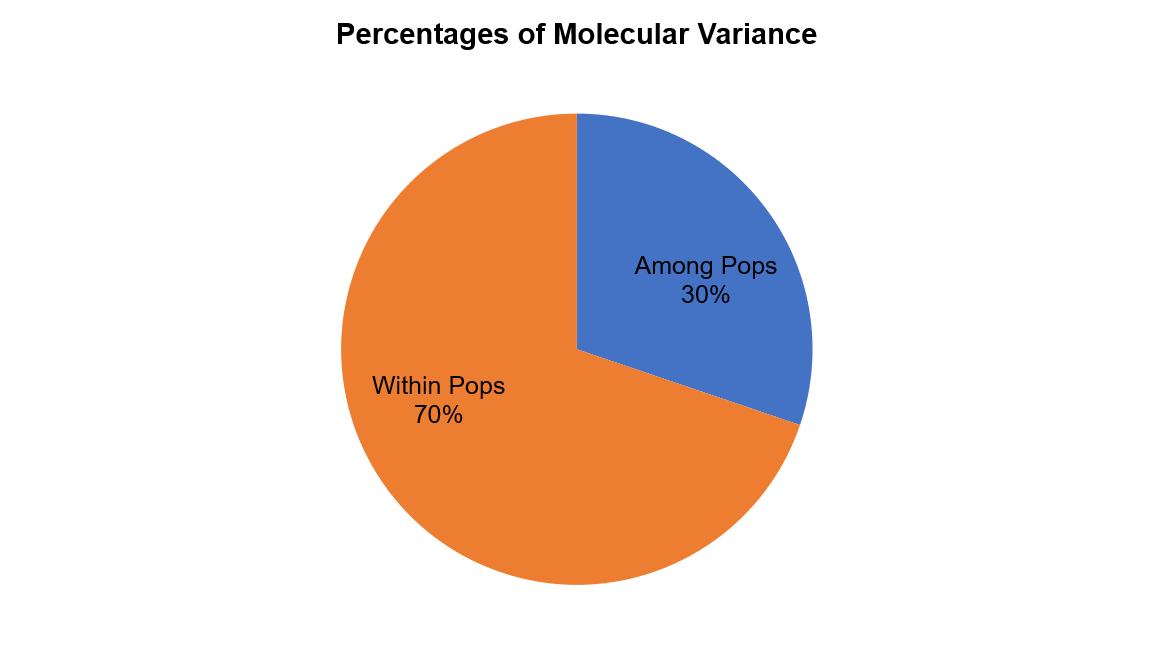

Supplement: S2 Fig — (TIF) [file pntd.0014472.s002.tif]

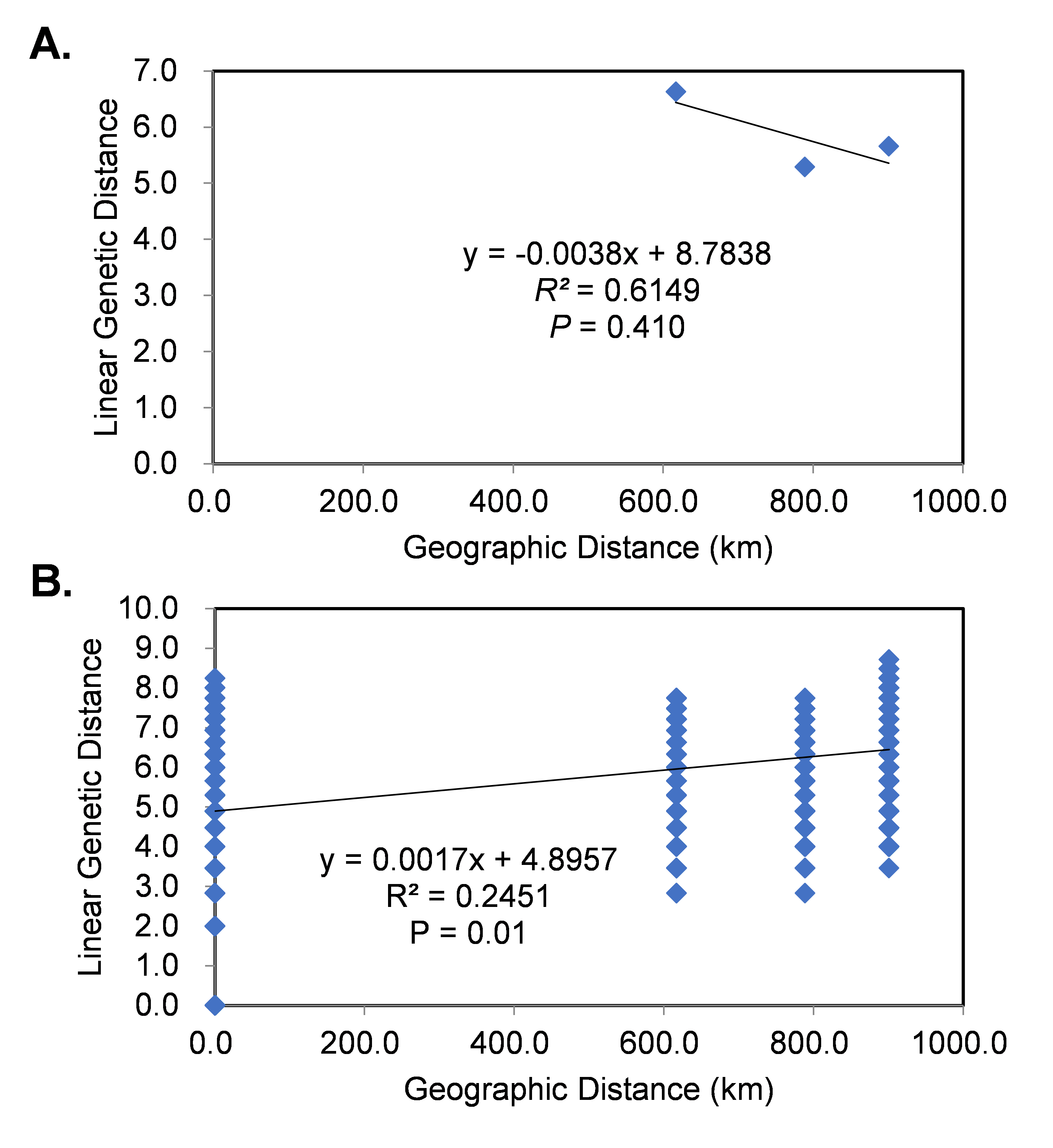

Supplement: S3 Fig — (A) Population-level analysis using the three sampling sites as independent analytical units. (B) Individual-level analysis based on pairwise genetic and geographic distances. (TIF) [file pntd.0014472.s003.tif]

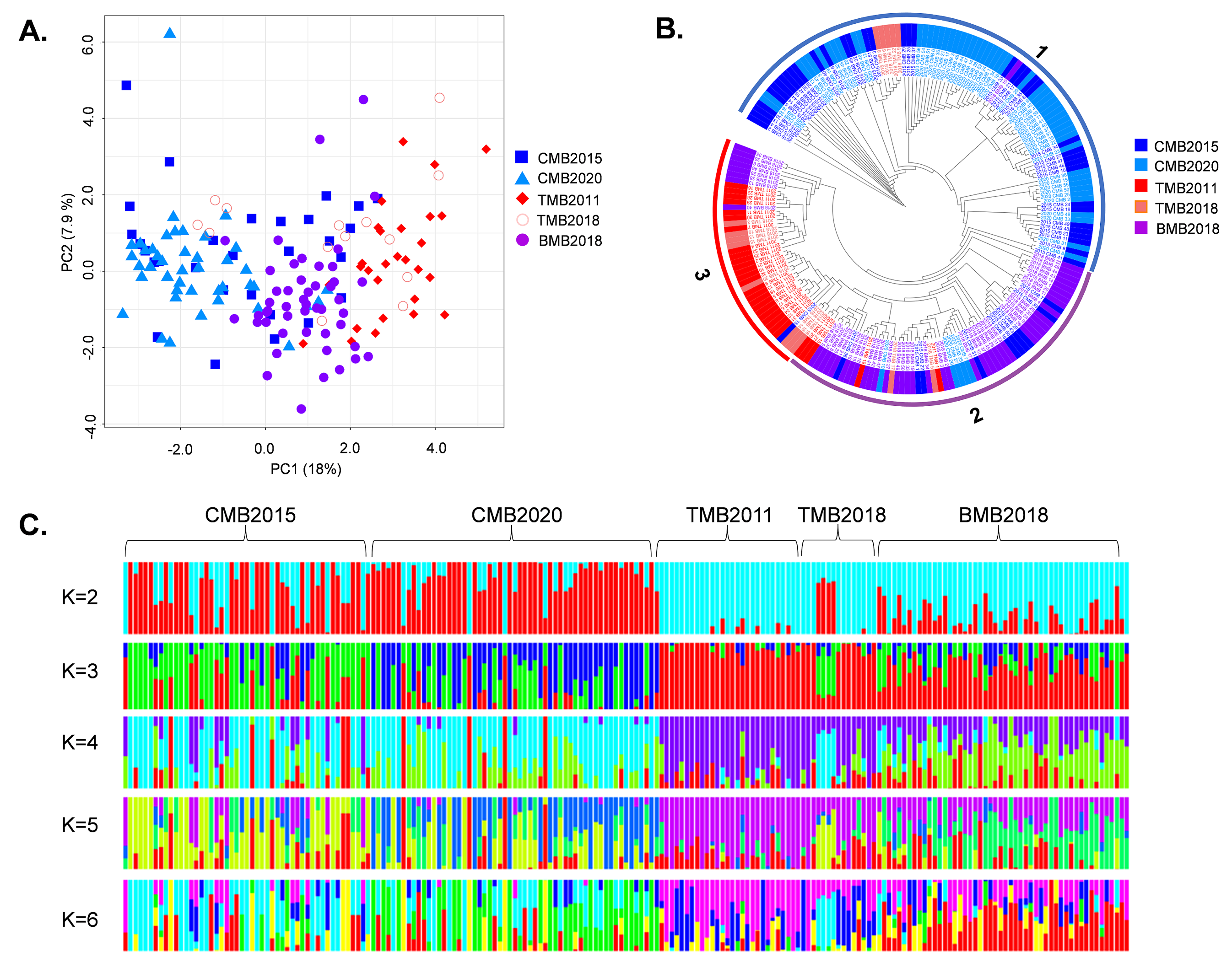

Supplement: S4 Fig — (A) Principal coordinate analysis. The 26 SNPs showed a similar power to distinguish P. vivax populations as the 22-SNP barcode. (B) The phylogenetic analysis using the neighbor-joining method. (C) ADMIXTURE analysis (K = 2 – 6). (TIF) [file pntd.0014472.s004.tif]
