# Supplementary material for: Spatiotemporal differentiation of Plasmodium vivax populations in the western Greater Mekong Subregion using a 22-SNP barcode
Source: PLoS Negl Trop Dis. 2026 Jun 29;20(6):e0014472. doi: 10.1371/journal.pntd.0014472 (PMC13340800; doi:10.1371/journal.pntd.0014472)
Supplement: S1 Table — (DOCX) [file pntd.0014472.s005.docx]

**S1 Table. 36-SNP molecular barcode and primers used in MassARRAY assay.**

| **Well** | **SNP_ID** | **2nd-PCRP amplification primer** | **1st-PCRP amplification primer** | **UEP_SEQ extended primers** | **UEP_DIR extension direction** |
| --- | --- | --- | --- | --- | --- |
| **W1** | SNP26 | ACGTTGGATGTAGCATAAGGCAAAAGGCCC | ACGTTGGATGTTCGTGAAACGGCAGTGTTG | CCCACCCCCAAGTGTAA | R |
| **W1** | SNP03 | ACGTTGGATGCCGTAAGACAAATCTGGGTG | ACGTTGGATGAACTGGTTGGTAAAGCCCTC | ATCGAGTAGGTCCCCTT | R |
| **W1** | SNP05 | ACGTTGGATGACTTCGACGGAAAAAGGCTC | ACGTTGGATGAGGTATTTTCTGTCATCCTC | TGGAATTGATCCCTTCTT | F |
| **W1** | SNP25-1 | ACGTTGGATGCGATCATGCTGGTCACAAAG | ACGTTGGATGTGGGTTTGTTGGCTGATTGG | gGTACCATGCGGGGAACC | R |
| **W1** | SNP09 | ACGTTGGATGGAACAGCAAGTCCATGTACG | ACGTTGGATGTCGCCATGTGATCTACTCTG | ACGAGGATGAGTTCTGGG | F |
| **W1** | SNP15 | ACGTTGGATGTGAATCCGTTTCCGCTAACC | ACGTTGGATGGAAGCAATGGAATGTGTGGG | agTCCGCTAACCACGTCAA | F |
| **W1** | SNP29 | ACGTTGGATGATGCTGACTGCTGTGTGATG | ACGTTGGATGCGTACAGCTCATCTCACCAC | CCCTCTCCCAGTTGTATTTA | R |
| **W1** | SNP28 | ACGTTGGATGAGCCCCAGCATTGCCATGAA | ACGTTGGATGTGTGCGGGTTGCAGCTTCT | TTGCCATGAACAGCTGCAAT | R |
| **W1** | SNP27 | ACGTTGGATGTACTCAAGCCGTGTACTTCC | ACGTTGGATGGCAGCTGCAGTTGCATGTTT | ggacCCCGTGCGTTTGTGTGA | R |
| **W1** | SNP23 | ACGTTGGATGCGTCAATGTCATGACTCCTG | ACGTTGGATGCATATGATCCATCACGTGGG | aggCAGACGAAAATGTGAGTT | R |
| **W1** | SNP08 | ACGTTGGATGTCATCACGGCCAACGACTTC | ACGTTGGATGATGGCCGCCCTTCTCCTATA | cccctCAGCTCCGCGATTACGA | R |
| **W1** | SNP21 | ACGTTGGATGCTAGATGGGTTCTTCTCTCC | ACGTTGGATGTGGTTAGACCTGTTAGGAAG | tctTCTTTATGTGCAAATCGAC | F |
| **W1** | SNP24 | ACGTTGGATGCTGTACCTCTCGGACAAATC | ACGTTGGATGCGACATGCTGAAGGATAACC | ggagGGCGCACAGGGTCTCGAA | R |
| **W1** | SNP22 | ACGTTGGATGCCAGGGAAACCTGTATCTTC | ACGTTGGATGATGGGCAAAGGCATGAACAC | AATCACGTCCTTATGAGATATGA | F |
| **W1** | SNP19 | ACGTTGGATGACAGTTGAAGCTACTGCTGG | ACGTTGGATGCTACCTGAGTGAGAGGAAAG | cttgGCCTCTCCCTGCTTGACTAC | F |
| **W1** | SNP20 | ACGTTGGATGCTACAACCAGCGAGAGTATG | ACGTTGGATGGGAAGTTAGAGGGTGTGTGG | cttaGCTATCTTATCCGGACATAT | R |
| **W1** | SNP17 | ACGTTGGATGTTTTACTGAGAAGGAAAGG | ACGTTGGATGGACAAGCGAAATGATTTGGG | gtgcGAAGGAAAGGTATACTCTCT | R |
| **W1** | SNP16 | ACGTTGGATGTTTGGCGTCACCTTCCAATG | ACGTTGGATGGCAAAAGCTAGGAAAGCTAC | cctgCCAATGGTTTATCTTTATCGG | F |
| **W1** | SNP14 | ACGTTGGATGCCATATTGCCGCTATTCGAG | ACGTTGGATGGGGAACGAAAAATGGAGAGG | ggatTGTCCAAGCAGATCGTTATTA | F |
| **W1** | SNP01 | ACGTTGGATGCAAAATTATGATCGGAAGC | ACGTTGGATGACTTCGGCAATTATAGGTTC | gacTTATGATCGGAAGCAAATTTTA | R |
| **W2** | SNP35 | ACGTTGGATGTTCTGCGCAGAAGCAGTACC | ACGTTGGATGGGTTCACCTGGTGGATCTC | GCCACTACAGCTTAGTT | F |
| **W2** | SNP18 | ACGTTGGATGAATGCTGGCTGTGAGCATTC | ACGTTGGATGAAGATAAAGCAGGGGAGTGG | GTGAGCATTCGCGTGGT | F |
| **W2** | SNP34 | ACGTTGGATGAAATGCGCCTACTGTGAGTG | ACGTTGGATGATACACTCAGCCAGAGTGCG | TGCATACGGCGCTAACTT | F |
| **W2** | SNP04 | ACGTTGGATGTAGGGCGTTTTTTGCGAAGG | ACGTTGGATGACACCTCGCACAGGGATTTT | gCCTTGGGAAGATGAGGA | R |
| **W2** | SNP31 | ACGTTGGATGCGGAGAGGTCTTTTCCATAC | ACGTTGGATGGGCGGGATATAACGACTTAG | TACAGTGGGGTATCCCAAA | F |
| **W2** | SNP11 | ACGTTGGATGAGAGCGACCCATTCATCACG | ACGTTGGATGTCCACCAGTTTGCGAACACG | AATTCTCATTCCGGTATTTC | F |
| **W2** | SNP10 | ACGTTGGATGATGGACATATGCACGTGGAC | ACGTTGGATGGTGTGTATGAGTTGTGCGTC | CGTGGACATATACACACAGA | F |
| **W2** | SNP30 | ACGTTGGATGATAGCGACGATGTCGTCCAG | ACGTTGGATGACACTCTTGTTGCTCACCCG | ggccGGCTCTACGCGCGCCTG | R |
| **W2** | SNP06 | ACGTTGGATGTTTCCCATACGAGGCAATCC | ACGTTGGATGTCTTCCTCTTCGGTGCTGG | caACGAGGCAATCCTCTTGTG | F |
| **W2** | SNP32 | ACGTTGGATGTCATCGAAGGCTTCAAGCTG | ACGTTGGATGCGAGTAGGTTCGTATTAGCC | gAGCTCGCCGCGCAAAAAGGG | R |
| **W2** | SNP13 | ACGTTGGATGGAGATGCTATACATTTATTG | ACGTTGGATGATAGTTAAAACATATCACAG | ccccACTAAATCCAGCGACTTA | F |
| **W2** | SNP02 | ACGTTGGATGTGGCAAACGAACGTAACCAC | ACGTTGGATGTGAGTGAAGTGGGTAAGCAG | gggaGTAACCACCTTGGTGAGT | F |
| **W2** | SNP25-2 | ACGTTGGATGCGATCATGCTGGTCACAAAG | ACGTTGGATGTGGGTTTGTTGGCTGATTGG | cccctTGTACCATGCAGGGAACC | R |
| **W2** | SNP12 | ACGTTGGATGCCTGAAATACGATAAGACGAC | ACGTTGGATGCGCAATTGTACGTATCTGCC | ACGATAAGACGACTTTCTACAAA | F |
| **W2** | SNP36 | ACGTTGGATGTCGAGTCGCAGAAGATGAAG | ACGTTGGATGTATATGCACACGCGTACGTC | gGAAGAAGTACATCGATAACAAC | F |
| **W2** | SNP33 | ACGTTGGATGTTCTCTTTGATGTCGGCCTG | ACGTTGGATGGGGTACTCATCGTAAACGTG | gggttATGTCGGCCTGGGGTTTG | R |
| **W2** | SNP07 | ACGTTGGATGGTAGGAACAACTCAAACAGC | ACGTTGGATGAATAAGTGATAGATAGTCC | CAGCAAAGAAATAAATCAATGCAAT | F |
