# Supplementary material for: Spatiotemporal differentiation of Plasmodium vivax populations in the western Greater Mekong Subregion using a 22-SNP barcode
Source: PLoS Negl Trop Dis. 2026 Jun 29;20(6):e0014472. doi: 10.1371/journal.pntd.0014472 (PMC13340800; doi:10.1371/journal.pntd.0014472)
Supplement: S2 Table — (DOCX) [file pntd.0014472.s006.docx]

**S2 Table. The amplification and sequencing primer sequences used for SNP assay** **1, 7, 13 and 21 on an ABI 3730XL DNA analyzer.**

| **SNP assay** | **Forward primer (5’ – 3’)** | **Reverse primer (5’ – 3’)** | **Product (bp)** |
| --- | --- | --- | --- |
| **SNP01** | CATTTGTCGAAACCGTCAGG | AAGCAAAATGGGGAGAATAC | 153 |
| **SNP07** | GCACCATGTAGGAACAACTC | CACTTATTTTTGTTTCGTG | 131 |
| **SNP13** | GTTATAACTTCTTTGTGACG | ATAGTTAAAACATATCACAG | 157 |
| **SNP21** | CTAGATGGGTTCTTCTCTCC | TGGTTAGACCTGTTAGGAAG | 109 |
