# Supplementary material for: Spatiotemporal differentiation of Plasmodium vivax populations in the western Greater Mekong Subregion using a 22-SNP barcode
Source: PLoS Negl Trop Dis. 2026 Jun 29;20(6):e0014472. doi: 10.1371/journal.pntd.0014472 (PMC13340800; doi:10.1371/journal.pntd.0014472)
Supplement: S3 Table — Red font indicates that the total MAF value is less than 0.05. WC, western China; NEM, northeastern Myanmar; WM, western Myanmar; SM, southern Myanmar; WT, western Thailand. (DOCX) [file pntd.0014472.s007.docx]

**S3 Table.** **Minor allele frequency (MAF) of *P. vivax* populations**.

| **SNP assay** | **Chrom.** | **SNPs** | **MAF (Total)** | **CMB2015** | **CMB2020** | **TMB2011** | **TMB2018** | **BMB2018** |
| --- | --- | --- | --- | --- | --- | --- | --- | --- |
| **SNP01 ^a^** | 1 | 907060 | - | - | - | - | - | - |
| **SNP02 ^b^** | 2 | 234032 | 0.005 | 0.000 | 0.009 | 0.000 | 0.000 | 0.000 |
| **SNP03 ^b^** | 2 | 301614 | 0.005 | 0.000 | 0.000 | 0.000 | 0.000 | 0.020 |
| **SNP04** | 4 | 435299 | 0.457 | 0.286 | 0.412 | 0.293 | 0.107 | 0.408 |
| **SNP05** | 5 | 1071362 | 0.295 | 0.265 | 0.167 | 0.190 | 0.143 | 0.418 |
| **SNP06** | 5 | 1087230 | 0.071 | 0.153 | 0.063 | 0.086 | 0.000 | 0.010 |
| **SNP07** **^a^** | 5 | 198537 | - | - | - | - | - | - |
| **SNP08** | 5 | 816823 | 0.331 | 0.296 | 0.079 | 0.293 | 0.107 | 0.500 |
| **SNP09** | 6 | 589259 | 0.316 | 0.102 | 0.018 | 0.069 | 0.500 | 0.459 |
| **SNP10** | 6 | 667280 | 0.356 | 0.439 | 0.333 | 0.000 | 0.357 | 0.000 |
| **SNP11** | 7 | 373079 | 0.240 | 0.163 | 0.026 | 0.310 | 0.321 | 0.173 |
| **SNP12** | 8 | 595633 | 0.189 | 0.061 | 0.000 | 0.448 | 0.321 | 0.245 |
| **SNP13** | 9 | 2162147 | 0.155 | 0.122 | 0.102 | 0.397 | 0.231 | 0.082 |
| **SNP14** | 9 | 431426 | 0.442 | 0.367 | 0.395 | 0.138 | 0.071 | 0.347 |
| **SNP15** | 10 | 1439810 | 0.404 | 0.418 | 0.219 | 0.034 | 0.357 | 0.020 |
| **SNP16** **^b^** | 10 | 1441770 | 0.000 | 0.000 | 0.000 | 0.000 | 0.000 | 0.000 |
| **SNP17** | 10 | 446044 | 0.449 | 0.367 | 0.263 | 0.138 | 0.357 | 0.135 |
| **SNP18 ^b^** | 11 | 1708471 | 0.005 | 0.000 | 0.009 | 0.017 | 0.000 | 0.000 |
| **SNP19** | 12 | 1098224 | 0.228 | 0.092 | 0.018 | 0.397 | 0.357 | 0.347 |
| **SNP20** | 12 | 1552911 | 0.126 | 0.122 | 0.298 | 0.034 | 0.000 | 0.020 |
| **SNP21** **^a^** | 12 | 270635 | - | - | - | - | - | - |
| **SNP22** | 12 | 334571 | 0.225 | 0.194 | 0.105 | 0.379 | 0.500 | 0.082 |
| **SNP23 ^b^** | 12 | 353253 | 0.000 | 0.000 | 0.000 | 0.000 | 0.000 | 0.000 |
| **SNP24** | 13 | 1024779 | 0.109 | 0.000 | 0.079 | 0.362 | 0.250 | 0.061 |
| **SNP25 ^c^** | 13 | 1079191 | 0.023 | 0.000 | 0.000 | 0.052 | 0.071 | 0.042 |
| **SNP26** | 13 | 1395659 | 0.366 | 0.306 | 0.404 | 0.086 | 0.393 | 0.153 |
| **SNP27** | 13 | 1696786 | 0.490 | 0.286 | 0.368 | 0.172 | 0.357 | 0.245 |
| **SNP28 ^c^** | 13 | 455190 | 0.010 | 0.000 | 0.000 | 0.000 | 0.143 | 0.000 |
| **SNP29** | 13 | 475776 | 0.417 | 0.306 | 0.360 | 0.103 | 0.357 | 0.327 |
| **SNP30** | 13 | 906341 | 0.199 | 0.000 | 0.054 | 0.321 | 0.214 | 0.286 |
| **SNP31 ^c^** | 14 | 1231865 | 0.030 | 0.000 | 0.088 | 0.000 | 0.000 | 0.020 |
| **SNP32** | 14 | 1255358 | 0.327 | 0.296 | 0.063 | 0.121 | 0.500 | 0.286 |
| **SNP33** | 14 | 1270401 | 0.495 | 0.429 | 0.268 | 0.034 | 0.357 | 0.500 |
| **SNP34 ^c^** | 14 | 2173639 | 0.031 | 0.000 | 0.115 | 0.000 | 0.000 | 0.000 |
| **SNP35 ^b^** | 14 | 2586551 | 0.000 | 0.000 | 0.000 | 0.000 | 0.000 | 0.000 |
| **SNP36** **^b^** | 14 | 992879 | 0.000 | 0.000 | 0.000 | 0.000 | 0.000 | 0.000 |

^a^ Sites with a detection rate of less than 90%; ^b^ The total MAF value is less than 0.05; ^c^ The total MAF value is less than 0.05, whereas the MAF in any specific region exceeds 0.05. CMB, China-Myanmar border; TMB, Thailand-Myanmar border; BMB, Bangladesh-Myanmar border.
